# Are personality traits associated with smoking and alcohol use prior to and during pregnancy?

**DOI:** 10.1371/journal.pone.0232668

**Published:** 2020-05-18

**Authors:** Magdalena Leszko, Lauren Keenan-Devlin, Emma K. Adam, Claudia Buss, William Grobman, Hyagriv Simhan, Pathik Wadhwa, Daniel K. Mroczek, Ann Borders

**Affiliations:** 1 Department of Psychology, University of Szczecin, Szczecin, Poland; 2 Division of Maternal Fetal Medicine, Department of Obstetrics and Gynecology, NorthShore University Health System, University of Chicago Pritzker School of Medicine, Evanston, IL, United States of America; 3 Institute for Policy Research, Evanston, IL, United States of America; 4 School of Education and Social Policy, Northwestern University, Evanston, IL, United States of America; 5 Health and Disease Research Program, University of California Irvine, Irvine, CA, United States of America; 6 Charité, Universitätsmedizin Berlin, Berlin, Germany; 7 Division of Maternal-Fetal Medicine, Department of Obstetrics and Gynecology, Northwestern University Feinberg School of Medicine, Evanston, IL, United States of America; 8 Center for Healthcare Studies, Institute for Public Health and Medicine, Chicago, IL, United States of America; 9 Division of Maternal-Fetal Medicine, University of Pittsburgh School of Medicine, Pittsburgh, Pa, United States of America; 10 Division of Obstetrical Services, Magee Women’s Hospital, Pittsburgh, Pa, United States of America; 11 Department of Psychology, Northwestern University, Weinberg College of Arts & Sciences, Evanston, IL, United States of America; University of Oslo, NORWAY

## Abstract

Cigarette smoking and alcohol consumption during pregnancy can have detrimental effects on the developing fetus, including fetal alcohol syndrome and low birth weight. Surprisingly little is known about the association of personality traits with smoking and alcohol consumption in the specific subpopulation of pregnant women. This study analyzed data from a geographically diverse sample of 603 pregnant women, aged 18 years and older, who provided information regarding their smoking and drinking habits before and during pregnancy. We compared women who consumed alcohol or smoked cigarettes before pregnancy with women who quit or continued smoking or drinking during pregnancy. Associations between personality and maladaptive behaviors prior to and during pregnancy were modeled using logistic regression. The study revealed that women who scored high on openness to experience were significantly more likely to continue alcohol consumption during pregnancy (OR = 1.07, 95% CI 1.01, 1.14, *p* = .02). This association was maintained after adjusting for potential confounds. This study demonstrated a significant relationship between personality traits and women’s likelihood of continued alcohol consumption prior to and during pregnancy. Understanding personality-based determinants of health-detrimental behavior is important in order to design interventions that aim at decreasing rates of maladaptive health behaviors among pregnant women.

## Introduction

The rationale for encouraging pregnant women to avoid smoking and consuming alcohol is compelling. One largely unexplored avenue for identifying who is at risk for these detrimental behaviors is the identification of personality traits. Although these traits are well-known predictors of smoking and excessive drinking, they have not been well studied in the context of pregnancy. In this study, we examined these associations within a large sample of pregnant women.

There is strong evidence that smoking and alcohol consumption pose adverse short and long-term risks for pregnancy outcomes. Smoking during pregnancy is associated with an increased risk of Sudden Infant Death Syndrome (SIDS), decreased birth-weight, placental abruption, preterm premature rupture of membranes (PPROM), and pre-term labor [1–5]. The effects of maternal smoking are also associated with motor, sensory, and cognitive deficits in infants and toddlers [6]. The harmful effects of smoking during pregnancy continue into late childhood and even adulthood. A systematic review of cross-sectional and longitudinal studies revealed that there is an association between maternal smoking during pregnancy and an increased risk for attention deficit hyperactivity disorder (ADHD) and developing adolescent-onset drug dependence [7]. The magnitude of the harm caused by alcohol consumption during pregnancy is also staggering. The most severe consequences associated with alcohol consumption include an increased risk for miscarriage, stillbirth, fetal alcohol spectrum disorder (FASD), alcohol-related neurodevelopmental disorder (ARND), and low birth-weight [8, 9]. Alcohol consumption during pregnancy can also lead to a range of adverse lifelong physical, behavioral, and intellectual outcomes in children, such as poor coordination, hyperactive behavior, difficulty with attention, poor memory, and speech and language delays [10]. Even low levels of alcohol consumption (less than one drink per week) may cause adverse neurobehavioral effects [11, 12]. Therefore, the American Congress of Obstetricians and Gynecologists (ACOG), the American Academy of Pediatrics, and other national and international medical societies (e.g., Centers for Disease Control and Prevention) recommend that women should avoid alcohol entirely while pregnant or trying to become pregnant [13,14].

Although warnings about the adverse impact of continued smoking and alcohol consumption have led to declining rates of these harmful behaviors in the United States, recent data show that about 8.4% of women smoked and 10% consumed alcohol at some point during pregnancy [13, 15, 16]. Researchers have been trying to identify factors that differentiate women who quit from those who continued to smoke or drink during pregnancy, despite the well-known health risks. Among the established predictors of continued smoking and drinking during pregnancy are certain sociodemographic characteristics such as lower levels of education, higher parity, being single, and lower levels of social support [17, 18]. Because cigarette smoking and alcohol use remain common modifiable risk factors for adverse pregnancy and birth outcomes [19], there is a need to better understand the determinants of continued smoking and drinking beyond demographic factors. A substantial body of literature suggests that personality traits, which comprise individual variation in behavioral tendencies, are potentially useful as predictors and may guide efficient intervention [20, 21].

Personality refers to individual differences in characteristic patterns of behaving, feeling, and thinking [22]. Although personality has been conceptualized in different ways, the Five Factor Model (FFM) has become a commonly accepted and reliable representation of personality. According to this model, the structure of the personality trait domain can be encompassed by five major dimensions: neuroticism (vs. emotional stability), extraversion (vs. introversion), openness to experience (vs. closeness to experience), conscientiousness (vs. lack of direction), and agreeableness (vs. antagonism) [23]. There have been a number of cross-sectional and longitudinal studies that have investigated the relationship between personality and various health behaviors in the general population. Many studies have shown that personality is an important predictor of cigarette smoking and alcohol consumption.

Neuroticism was found to be associated with an increased likelihood of smoking [24–26]. Individuals who scored high on instruments used to detect this trait tend to struggle with emotional control, more often display negative affect such as sadness, depression, and anxiety [23] and, consequently, may smoke to reduce stress and feelings of worry [27]. In addition, smokers with elevated levels of neuroticism tend to smoke more and have greater difficulty quitting [20, 28–30]. The evidence for a relationship between high neuroticism and excessive drinking is more equivocal, but some evidence demonstrates that higher neuroticism is related to alcohol abuse and dependence [28, 31–34]. Similar to those who smoke, individuals with greater levels of neuroticism may use alcohol as a way to self-medicate feelings of anxiety [25].

A number of studies have shown that extraversion, which is associated with larger social networks and increased social interaction [e.g., 34, 35, 36] is positively associated with smoking [29, 37–39] and alcohol consumption [21, 40]. Some researchers have noted that the association between smoking and extraversion has decreased in recent decades [41–43]. One of the possible explanations is that smoking has become a non-normative behavior as more people have become aware of the adverse consequences of smoking [44]. Research has demonstrated that individuals who score high on extraversion initiate alcohol use at an earlier age [45, 46] and tend to consume more alcohol [47]. In addition, individuals high in extraversion derive more mood-enhancing effects from alcohol than those with low levels of extraversion [48].

Conscientiousness and aspects of conscientiousness are also associated with smoking and alcohol consumption. Individuals with high levels of conscientiousness are more self-disciplined, hardworking, dutiful, reliable, and achievement-oriented [23], tending to be careful about taking action that could possibly damage their reputation [49]. In a meta-analysis, Malouff and colleagues [33] found that alcohol consumption was higher in individuals with low conscientiousness. Bogg and Roberts [50] found that low conscientiousness was associated with excessive alcohol use. Low conscientiousness has been also found as a risk factor for smoking. Results of a longitudinal study revealed that low conscientiousness in children was a predictor of smoking in adulthood [51].

The findings on other personality traits (agreeableness and openness to experience) as related to smoking and alcohol consumption are less consistent. While some researchers reported that low agreeableness is associated with an increased chance of being a smoker [20, 52, 53], the meta-analysis by Hakulinen and colleagues [54] of nine cohort studies from Australia, Germany, the UK, and the US (n = 79,757) did not find this association. Two large meta-analyses investigating the associations between personality traits and alcohol consumption found that low agreeableness is associated with alcohol consumption, [33, 55] whereas individuals high on this trait are more likely to quit drinking or abstain from alcohol [55].

The findings regarding openness to experience and maladaptive behaviors are also largely inconsistent [56]. Some individual studies have found that openness is associated with binge drinking among college women [57], but other studies also conducted on college females found the opposite- binge drinkers rated lower in openness than non-binge drinkers [58]. Rush et al. [59] did not find that openness was associated with drinking when conducting research on both male and female college students. One meta-analysis found that low openness to experience was associated with abstinence or a decrease in alcohol consumption [60]. While some studies suggest the association between high openness to experience and smoking [56, 61], other studies did not find this association [20].

Given these existing findings in the general population, there is reason to suspect that personality traits may also predict smoking and drinking behaviors among women during pregnancy. The associations between maladaptive behaviors and personality traits in a population of pregnant women may be stronger than those of the general population. Research has shown that new roles, such as becoming a mother, are related to certain social expectations and adaptive processes which in turn may lead to changes in personality such as an increase in conscientiousness and a decrease in neuroticism [62, 63]. On the other hand, pregnancy is a unique period in a woman’s life in which they are highly motivated to quit maladaptive behaviors and this factor may have a greater impact on changing behavior than is attributable to personality traits. Nevertheless, understanding the association between personality traits and maladaptive behaviors is crucial and may offer insights into how to design effective interventions for pregnant women.

To our knowledge, very few studies have addressed the issue of personality traits and maladaptive behaviors among pregnant women. Beijers and colleagues [64] found that higher levels of openness to experience and lower levels of conscientiousness among pregnant women were associated with alcohol consumption, but none of the personality traits were associated with continued smoking. Using the same measure of personality, NEO-FFI, Maxon and colleagues [65] found that low levels of agreeableness were associated with continued smoking. Looking specifically at smoking status before and during pregnancy, Massey and colleagues [66] found that women with a tendency to worry in anticipation of future problems were more likely to continue smoking during pregnancy. Low self-directedness, which can be expressed by an inability to delay immediate gratification, was also associated with smoking during pregnancy. The differences in findings may be due to different sample sizes, measures of personality and the retrospective nature of the Massey et al. [66] study.

The current study examined the associations between personality and continued smoking and alcohol use prior and during pregnancy. Specifically, we analyzed differences among women who did not smoke nor consume alcohol prior, women who continued smoking or drinking, and women who quit these maladaptive behaviors. Based on the previous research reviewed above, we hypothesized that women with high levels of extraversion would be less likely to smoke cigarettes, mainly because smoking has become a socially undesirable habit, especially among pregnant women. Because pregnancy is a life event considered to be stressful by many women [67, 68] we hypothesized that individuals high on neuroticism would be more likely to continue smoking and drinking during pregnancy in order to cope with stress. Based on the findings that show that conscientiousness is positively related to all beneficial health-related behaviors [50], we expect that women with low conscientiousness would be more likely to continue smoking and drinking during pregnancy. The majority of previous studies compared women who quit maladaptive health behaviors (smoking or drinking) with those who continued these behaviors throughout their pregnancy. Few studies differentiate between women who never smoked, women who quit smoking during pregnancy, and women who continued smoking [66]. It has been suggested that women who quit smoking during pregnancy possess adaptive characteristics and report fewer depressive symptoms [69]. Therefore, in this study we also analyzed data from women who did not smoke or consume alcohol prior to pregnancy and compared them to women who either continued or quit maladaptive behaviors.

## Materials and methods

### Participants

The study population comes from the Measures of Maternal Stress (MOMS) Study, a sub-study of the National Children’s Study (NCS), which aimed to optimize the measurement of stress and key correlates of stress in women during pregnancy. Women were enrolled from prenatal clinics as part of a multisite prospective cohort study that included four geographically and racially diverse regions (Pittsburgh, PA, Chicago IL, Schuylkill County PA, and San Antonio TX) between June 2013 and May 2015. Detailed information about the study has been provided in previous reports [70, 71]. Eligible participants were 18 years of age or older, carrying a singleton pregnancy, English-speaking, and had no evidence of fetal, congenital, or genetic anomalies. Detailed information on the socio-economic, health, and demographic characteristics of the study population used in this analysis was collected via survey and bio sample collection between 12 and 20 weeks of gestation, and after delivery via postpartum medical chart review. Of the 744 participants, 603 provided complete data on the key variables (smoking, alcohol consumption, and personality traits). Attrition analyses revealed that respondents who were excluded from the analysis (n = 141) did not differ significantly from those who were included on demographic variables. Prior to data collection, ethics approval for the original data collection was provided by the Institutional Review Board of Northwestern University in Evanston, IL (project number STU00039484). All participants signed written informed consent and received 40 U.S. dollars for their participation. The current study is a secondary analysis of de-identified data.

### Measures

#### Smoking status

The dichotomous dependent variable, cigarette smoking, was measured during the second trimester by asking each participant “In the 3 months before you became pregnant, did you use any cigarettes?” The answer was coded “1” for those who smoked and “0” for nonsmokers.

#### Alcohol consumption status

At the second trimester assessment, all participants were asked, “Did you drink alcohol before pregnancy?” If they responded “yes”, they were asked whether they consumed alcohol during pregnancy. The answer was coded “1” for those who consumed alcohol and “0” for those who stopped drinking.

#### Personality traits

Personality was measured using the NEO Five-Factor Inventory (NEO-FFI) [23]. The NEO-FFI is a self-report questionnaire consisting of 60 items answered on a five-point Likert scale ranging from 0 (strongly disagree) to 4 (strongly agree). The NEO-FFI is a shortened version of the NEO Personality Inventory (the NEO-PI-R) and assesses neuroticism, extraversion, conscientiousness, agreeableness, and openness to experience; each of the five subscales is comprised of 12 items. Respondents were given a list of statements such as “I like to have a lot of people around me” and “Too often, when things go wrong, I get discouraged and feel like giving up.” The summary score for each domain ranges from 0 to 48. NEO-PI-R scales have shown longitudinal stability, cross-observer agreement, and convergent and discriminant validity in a large body of studies [23, 72].

#### Measurement of covariates

Sociodemographic variables included maternal age, education, race/ethnicity, and number of previous births. Race/ethnicity was categorized as White, Black, Hispanic, and other. Educational attainment was categorized into four groups: “less than high school diploma”, “high school diploma or GED”, “some college”, and “Bachelor’s and higher”. Annual household income was reported in brackets: under $15,000; $15,000–$50,000; $50,000–$100,000; and more than $100,000.

### Statistical analyses

#### Descriptive statistics

The first step of the analysis was to investigate whether personality traits differed between groups of women who were not users of alcohol and cigarettes before pregnancy and those who continued or quit these behaviors during pregnancy. Comparisons of demographic characteristics were conducted using chi-square tests of independence for categorical variables. Missing categories (e.g., income or educational attainment) were included in all analyses in order to retain the full sample. To test differences in mean levels of personality traits between smoking and drinking categories, we performed a series of one-way analysis of variance (ANOVA). Then we followed up with post-hoc tests where appropriate. In order to further explore differences among women based on their smoking or drinking status, we conducted secondary comparisons using t-tests. The effect sizes were estimated with partial eta squares (η_p_^2^). According to Cohen [73], η^2^ values of .01, .06, and .14 correspond to small, medium, and large effect sizes, respectively.

#### Adjusted and unadjusted logistic regressions

In order to examine the relationship between personality traits and the likelihood of smoking and drinking before pregnancy, as well as the likelihood of continuing smoking or drinking, we carried out multiple logistic regressions, adjusting for demographic characteristics, including maternal age, race-ethnicity, education, income, and number of previous births. In each regression model, five factors of personality and demographic characteristics were included to examine the likelihood of each smoking and drinking outcome. We computed the odds ratios (OR) with a 95% confidence interval to estimate the risk for continuing smoking or drinking. A two-tailed alpha of 0.05 was used to define statistical significance. IBM SPSS software platform version 23.0 was used for statistical analyses [74].

## Results

### Sample characteristics

Table 1 presents the descriptive statistics for the personality scales used in the analyses grouped by smoking and drinking status. The sample for the current study was composed of 603 pregnant women who ranged in age from 18 to 50 years (*M* = 29.5, *SD* = 5.7). The sample was fairly well educated, 256 (42.6%) had attained a bachelor's or higher degree. Among all women, 381 (63.2%) were White, 80 (13.3%) were Black, and 110 (18.2%) were distributed across other racial categories. Of the total 603 women, smoking before pregnancy was reported by 143 (23.7%). More than half (n = 78, 55%) of these women reported that they had quit smoking after finding out about the pregnancy. Sixty-five (45%) of women reported that they had smoked during their pregnancy. Rates of alcohol consumption were higher: 440 (73%) women reported drinking before pregnancy. Of these women, 305 (69.3%) quit drinking after learning about the pregnancy and 44 (10%) continued to consume alcohol. Engaging in both smoking and consuming alcohol before pregnancy was reported by 110 (19% of women), and 37 (34%) of those who engaged in both behaviors prior to pregnancy reported continuing both smoking and drinking during pregnancy. None of the non-smoking and non-drinking women reported that they started smoking or consuming alcohol during pregnancy.

**Table 1:** Descriptive statistics of the study population.

		Smoked cigarettes				Consumed alcohol		
Variable	Did not smoke cigarettes	Quit smoking	Continued smoking	*p*-value*	Did not drink	Quit drinking	Continued drinking	*p*-value*
	(n = 460)^g^	(n = 78)	(n = 65)		(n = 123)	(n = 305)	(n = 44)	
Age, mean (SD)	30.1 (5.6)	27.3 (5.1)	27.8 (6.0)	< .05^a^^,^^b^	27.6 (5.5)	29.5 (5.5)	31.5 (5.6)	< .05^d^^,^^e^
Education, n (%)				< .05				< .05
Less than high school	26 (5.7)	7 (9.0)	12 (18.5)		15 (12.2)	21 (6.9)	1 (2.3)	
High school diploma or GED	54 (11.8)	28 (35.9)	20 (30.8)		36 (29.3)	47 (15.5)	7 (15.9)	
Some college	137 (29.9)	33 (42.3)	28 (43.1)		42 (34.1)	94 (30.9)	12 (27.3)	
Bachelor's Degree and higher	241 (52.6)	10 (12.8)	5 (7.7)		30 (24.4)	142 (46.7)	24 (54.5)	
Race or ethnicity, n (%)				< .05				.03
Non-Hispanic white	283 (65.4)	51 (67.1)	47 (75.8)		69 (60.5)	199 (69.1)	36 (83.7)	
Black	61 (14.0)	11 (14.5)	8 (12.9)		14 (12.3)	40 (13.9)	2 (4.7)	
Hispanic	89 (20.6)	14 (18.4)	7 (11.3)		31 (27.2)	49 (17.0)	5 (11.6)	
Income, n (%)				< .05				< .05
<15k	44 (10.1)	17 (22.3)	21 (38.9)		23 (21.7)	39 (13.8)	4 (9.5)	
15-50k	125 (29.3)	30 (42.9)	22 (40.7)		47 (44.3)	82 (29.0)	9 (21.4)	
50-100k	136 (31.9)	19 (27.1)	10 (18.5)		28 (26.4)	85 (30.0)	16 (38.1)	
>100k	122 (28.6)	4 (5.7)	1 (1.9)		8 (7.5)	77 (27.2)	13 (31.0)	
Number of previous births				.06				.02
Nulliparous	209 (45.5)	42 (53.8)	22 (33.8)		42 (34.1)	145 (47.5)	23 (52.3)	
Multiparous	250 (54.5)	36 (46.2)	43 (66.2)		81 (65.9)	160 (52.5)	21 (47.7)	
Personality traits (M, SD)								
Neuroticism	20.5 (7.5)	22.3 (7.6)	24.0 (7.6)	< .05^a^^,^^b^	22.0 (7.0)	20.2 (7.9)	22.0 (7.0)	.06
Extraversion	29.2 (5.7)	27.5 (6.0)	26.8 (6.0)	< .05^a^^,^^b^	27.6 (5.4)	29.4 (5.9)	28.4 (6.6)	< .05^d^
Openness to experience	26.5 (5.9)	26.7 (5.0)	24.9 (6.0)	.08	25.2 (5.9)	26.5 (5.7)	29.1 (6.2)	< .05^e^^,^^f^
Conscientiousness	24.9 (3.2)	25.8 (3.0)	25.4 (3.1)	.08	25.4 (3.5)	24.9 (3.0)	25.2 (2.6)	.03
Agreeableness	32.9 (5.7)	30.8 (5.3)	31.3 (5.4)	< .05^a^	32.2 (5.2)	32.3 (5.7)	32.0 (5.3)	.09

M = mean; SD = standard deviation

*ANOVA or chi-square Test

^a^ *p* < .05 between women who did not smoke cigarettes and quit smoking

^b^ *p* < .05 between women who did not smoke cigarettes and continued smoking

^c^ *p* < .05 between women who quit smoking and continued smoking

^d^ p < .05 between women who did not drink alcohol and quit drinking

^e^ *p* < .05 between women who did not drink alcohol and continued drinking

^f^ *p* < .05 between women who quit drinking and continued drinking

^g^ Numbers may not add to totals or percents to 100 due to missing data.

### Differences between groups based on smoking status

Women differed significantly on almost all sociodemographic characteristics. Women who continued smoking were more likely to be White, less educated, had lower income, and more children compared to women who quit smoking. Based on smoking status, one-way ANOVAs revealed that there were significant differences in age and personality traits. The groups differed in age (*F*(2, 599) = 11.7, *p* < .001, η_p_^2^ = .038). Post hoc comparisons using the Tukey HSD test revealed that women who never smoked were significantly older than women who continued or quit smoking (*p* < .001, *p* = .005, respectively). There were statistically significant differences in neuroticism, *F*(2, 601) = 9.07, *p* = .001, η_p_^2^ = .031, extraversion, *F*(2, 599) = 6.58, *p* = .001, η_p_^2^ = .022, and agreeableness, *F*(2, 600) = 6.40, *p* = .002, η_p_^2^ = .021 among groups. Post hoc comparisons revealed that women who quit smoking and those who continued smoking during pregnancy had higher levels of neuroticism than women who did not smoke prior to their pregnancy (*p* = .04, *p* < .001, respectively). Women who did not smoke before pregnancy had higher levels of extraversion than women who quit smoking and those who continued smoking during pregnancy (*p* = .04, *p* = .001, respectively). Women who did not smoke had also higher levels of agreeableness than women who quit smoking during pregnancy (*p* = .001). Participants did not differ on the levels of openness to experience nor on the levels of conscientiousness.

### Differences between groups based on alcohol consumption

Women who continued drinking alcohol were more likely to be White, highly educated, to have high income, and fewer children than women who quit drinking alcohol during pregnancy. Women also differed significantly in regard to age, *F*(2, 469) = 9.88, *p* < .001, η_p_^2^ = .04. Women who continued drinking were significantly older than those who did not drink before pregnancy (*p* < .001). ANOVAs also indicated significant differences in levels of extraversion, [*F*(2, 469) = 4.37, *p* = .01, η_p_^2^ = .018] and openness to experience, *F*(2, 468) = 6.88, *p* = .001, η_p_^2^ = .028. Post hoc comparisons using the Tukey HSD test indicated that women who did not report consuming alcohol before pregnancy scored lower in levels of extraversion than women who quit drinking (*p* = .01). Women who continued drinking during pregnancy had significantly higher scores in openness to experience than women who never drank (*p* = .001) or who quit drinking (*p* = .026). Women who continued drinking reported higher openness to experience (M = 29.1, *SD* = 6.2) than women who quit drinking (*M* = 26.5, *SD* = 5.7), *t*(347) = -2.89, *p* = .004, *d* = .5, 95% CI = [-4.44, -.85]. No significant differences were found in the levels of agreeableness [*F*(2, 471) = 0.12, *p* = .89], neuroticism [*F*(2, 471) = 2.86, *p* = .058], nor conscientiousness [*F*(2, 463) = 1.07, *p* = .345].

#### Predictors of smoking prior to and during pregnancy

Table 2 shows the results of logistic regression analyses using smoking before pregnancy and during pregnancy as dependent variables. We ran an unadjusted model and a fully adjusted model that included all five personality traits as well as the demographic variables. Higher neuroticism and conscientiousness were associated with an increased likelihood of being a smoker prior to pregnancy (OR = 1.05; 95% CI 1.02, 1.08, p < 0.001; OR = 1.1; 95% CI 1.01, 1.1; *p* = .02, respectively), whereas higher agreeableness and extraversion were associated with lower likelihood of being a smoker (OR = 0.94; 95% CI 0.91, 0.97; *p* = .001; OR = 0.94, 95% CI 0.91, 0.97; p < .001, respectively.

**Table 2:** Associations between women’s personality and smoking before and during pregnancy.

Variable	Smoking before pregnancy^a^				Continuing to smoke during pregnancy^b^			
	unadjusted		adjusted*		unadjusted		adjusted*	
	OR (CI)	*p* value	OR	*p* value	OR (CI)	*p* value	OR (CI)	*p* value
Neuroticism	1.05 (1.02–1.08)	< .001	1.02 (0.99–1.05)	.18	1.03 (0.99–1.08)	.18	1.01 (0.96–1.06)	.61
Extraversion	0.94 (0.91–0.97)	< .001	0.97 (0.93–1.01)	.15	0.98 (0.93–1.04)	.52	1.02 (0.95–1.09)	.54
Openness	0.98 (0.95–1.01	.27	1.03 (0.98–1.07)	.14	0.94 (0.89–1.00)	.06	0.96 (0.89–1.04)	.37
Conscientiousness	1.1 (1.01–1.1)	.02	1.03 (0.95–1.1)	.46	0.96 (0.86–1.07)	.50	1.01 (0.88–1.1)	.83
Agreeableness	0.94 (0.91–0.97)	.001	0.95(0.91–0.99)	.03	1.02 (0.96–1.08)	.56	1.02 (0.94–1.01)	.62

OR = odds ratio.

*Model adjusted for demographic variables (income, parity, maternal age, education, race)

^a^Women who smoked before pregnancy compared to women who did not smoke before pregnancy

^b^Women who continued to smoke during pregnancy compared to women who quit smoking during pregnancy

In fully adjusted model analyses, only agreeableness remained a significant predictor of smoking before getting pregnant (OR = 0.95, 95% CI 0.91, 0.99 *p* = .03). Other predictors of smoking were low income and educational attainment. None of personality traits were associated with an increased likelihood of continuing to smoke during pregnancy.

#### Predictors of alcohol consumption prior and during pregnancy

As shown in Table 3, drinking alcohol before pregnancy was associated with lower levels of neuroticism and conscientiousness (OR = 0.97, 95% CI 0.94, 0.99, *p* = .02; OR = .095, 95% CI 0.83, 1.01, *p* = .002, respectively). Compared to the never drinking group, women who reported higher levels of extraversion and openness to experience were more likely to consume alcohol (OR = 1.05, 95% CI 1.01, 1.08, *p* = .004; OR = 1.04, 95% CI 1.01, 1.08, *p* = .009, respectively). However, these associations were no longer significant after adjusting for demographic variables. There was no association between agreeableness and alcohol consumption before pregnancy (see Table 3).

**Table 3:** Associations between women’s personality and alcohol consumption before and during pregnancy.

	Alcohol before pregnancy^c^				Continued alcohol consumption during pregnancy^d^			
Variable	unadjusted		adjusted*		unadjusted		adjusted*	
	OR (CI)	*p* value	OR	*p* value	OR (CI)	*p* value	OR (CI)	*p* value
Neuroticism	0.97 (0.94–0.99)	.02	0.97 (0.94–1.0)	.17	1.02 (0.98–1.06)	.19	1.04 (0.99–1.08)	.07
Extraversion	1.05 (1.01–1.08)	.004	1.03 (0.98–1.07)	.16	0.96 (0.91–1.01)	.20	0.96 (0.91–1.01)	.17
Openness	1.04 (1.01–1.08)	.009	1.02 (0.98–1.07)	.22	1.08 (1.02–1.1)	.005	1.07 (1.01–1.14)	.02
Conscientiousness	.095 (0.83–1.01)	.002	1.01 (0.94–1.09)	.63	1.07 (0.96–1.18)	.18	1.08 (0.96–1.22)	.18
Agreeableness	1.01 (.09–1.05)	.31	.097 (0.93–1.01)	.26	.097 (0.92–1.03)	.40	0.94 (0.88–1.00)	0.6

^c^Women who consumed alcohol before pregnancy compared to women who did not drink before pregnancy

^d^ Women who consumed alcohol during pregnancy compared to women who quit drinking

Openness to experience predicted increased odds of drinking during pregnancy (OR = 1.08, 95% CI 1.02, 1.1, *p* = .005). This personality trait remained a significant predictor even after controlling for demographic variables (OR = 1.07, 95% CI 1.01, 1.14, *p* = .02). None of the demographic variables emerged as a significant predictor of continued drinking.

## Discussion

In this large, diverse study of pregnant women we compared women who consumed alcohol or smoked cigarettes before pregnancy with women who quit or continued drinking or smoking while pregnant. The majority of women in this study quit smoking or drinking after learning about the pregnancy. However, 45% of women continued to smoke and 10% of women continued to consume alcohol during pregnancy.

Compared to non-drinkers, women who reported drinking before pregnancy and continued to drink were more likely to have high educational attainment and income. This confirms the findings of previous research which has demonstrated that individuals with higher socioeconomic status (SES) can be more likely to consume alcohol because of their greater economic resources. Higher SES not only allows individuals to buy alcohol but also to be exposed to it during social interactions (e.g., in restaurants) [75]. A growing body of research has also demonstrated that individuals with low SES are either more likely to abstain from consuming alcohol or to engage in heavy drinking whereas individuals with higher SES consume alcohol more frequently but in smaller amount [76]. Therefore, future research should address drinking frequencies and quantities to examine its relationship with sociodemographic correlates among pregnant women.

Contrary to women who consumed alcohol, women who smoked cigarettes and continued to smoke during pregnancy were more likely to be less educated and had lower income in comparison to non-smokers. A few meta-analyses have reported that lower education and income were consistently associated with higher smoking prevalence [77]. Perhaps less educated women are not aware of the detrimental effects of smoking on health. Another possible explanation is that women who continued smoking had struggled with withdrawal symptoms due to nicotine dependence.

In addition, women who continued to smoke while pregnant were more likely to already have had a child than women who continued to drink (66.2% vs. 47.7%, respectively). Prior research on smoking during pregnancy has documented that women who already have had a child were less likely to quit smoking than women who were pregnant for the first time [78, 79]. The prevalence of cigarette smoking among women with previous pregnancies can be higher if they did not experience complication but rather may have an experience of giving birth to healthy children [80], which in turn, may decrease their motivation to limit exposure to smoking.

While this study confirms the role of sociodemographic variables in predicting the likelihood of smoking and drinking during pregnancy, is it also one of the few studies that investigates the role of personality traits and maladaptive behaviors among a large group of pregnant women. Previous studies have shown that people with high levels of neuroticism and extraversion tend to consume more alcohol and smoke more cigarettes. We found that women with higher levels of extraversion were also more likely to use alcohol prior to pregnancy but were less likely to smoke. Although these results are contrary to some older findings where extraversion was strongly related to smoking, they confirm more recent findings that the association between extraversion and smoking has become weak in recent years [41–43]. This shift may reflect today's attitudes toward smoking and the raising awareness of the adverse consequences of smoking.

The results emerging from this study also indicate that higher levels of agreeableness were related to lower rates of smoking before pregnancy. This association persisted even after adjusting for demographic characteristics. We did not find any significant associations between personality traits and continued smoking during pregnancy.

The most important finding is that women with higher levels of openness to experience are more likely to continue drinking alcohol during pregnancy. This effect was observed even after correction for potential confounders, such as age, income, parity, and educational attainment. This finding was consistent with a recent study conducted in the Netherlands on a group of pregnant women [64] where high levels of openness to experience were also found to be associated with continued alcohol consumption during pregnancy. Interestingly, a relationship between openness to experience and alcohol consumption was found only in samples on pregnant women and other studies in the U.S. general population failed to confirm this association [54]. This may suggest that openness to experience plays an important role in reducing maladaptive behaviors among pregnant women. Further research is necessary to understand these discrepancies.

One of the possible explanations for an association between this particular personality trait and alcohol consumption, suggested by Massey and colleagues [66], is that women who score high on openness to experience get bored easily, are impulsive, and initiate smoking and drinking earlier than individuals with lower levels of this trait. In addition, this trait has been associated with both a predisposition to developing drug dependence and more difficulty in sustaining abstinence from alcohol. Perhaps higher levels of alcohol consumption among women who continued to drink during pregnancy reflect openness-related addictive tendencies and a struggle to abstain from alcohol during pregnancy [66, 81]. Another possible explanation is that women who are high on openness to experience have difficulty adhering to social norms and values and may question their doctors’ recommendations to abstain from alcohol [50]. This corresponds with the tendency for those scoring high on openness to experience to think and act in individualistic and nonconforming ways and to question authority [23].

This study was not without limitations. First, we relied on self-reported measures of smoking and alcohol consumption. Previous studies have demonstrated that smoking and alcohol consumption is often underreported, especially among pregnant women [82]. The analysis also lacks the frequency of smoking and drinking. In our study, participants who indicated that they smoked or consumed alcohol during pregnancy were then asked for a rating of the frequency of smoking cigarettes and drinking alcohol per day. Due to a high proportion of missing data regarding the frequency of smoking and drinking, we excluded these variables for the current analyses. Researchers point out that participants often refuse to answer a certain test item, therefore the problem of missing data regarding frequency of smoking and drinking is common in research studies. One of the possible explanations why participants refrain from reporting having engaged in such behavior may be a fear of reprisal. Thus, it is the number of cigarettes smoked that is causing a nonresponse on the smoking frequency item [83]. Pregnant women likely underreport maladaptive behaviors because most of them are aware that smoking and drinking is stigmatized during pregnancy, or as Wong and Koren [84] suggest, underreporting has been associated with maternal guilt. Our study is also limited by its cross-sectional design. It would be interesting to see if women who quit drinking or smoking returned to their habit after giving birth. DiClemente and colleagues [85] found that close to half of smokers suspend smoking over the many months of gestation without treatment, then resume smoking postpartum. Hannöver and colleagues [86] reported that the majority of those who quit returned to smoking within 12 months. Pregnancy provides a unique window of opportunity to quit smoking and drinking, and many women are highly motivated to do so [68]. A relapse of habits after delivery can negatively impact a woman’s health as well as expose her child to the environmental tobacco smoke [88]. A longitudinal study designed to observe which individuals return to smoking and drinking would extend the current line of research and provide a broader picture of the relationship between personality and maladaptive behaviors. It would also offer insights to support relapse-prevention strategies. Furthermore, the study did not assess levels of nicotine or alcohol dependence. Some studies demonstrated that the degree of tobacco or alcohol addiction is an important factor in predicting difficulties in quitting smoking and alcohol consumption [18, 87, 88, 89].

Nevertheless, we have demonstrated the link between certain personality traits and the likelihood of alcohol consumption before and during pregnancy. Given the potential harm of smoking and alcohol consumption on the woman and her child, it is of growing importance to prenatal and pediatric health care providers, midwives, and public health officials to learn more about the role of personality in decreasing the rates of continued smoking and drinking during pregnancy.

In summary, the findings provide important information that can be used in the design of interventions to decrease maternal smoking and alcohol consumption. It is clear that some women will quit on their own but others will need assistance [76]. Therefore, personality measures might be used as part of a general battery of assessments that index the risk of continued smoking or alcohol consumption post-pregnancy. Personality-informed risk assessment may be useful in determining how to tailor resources with respect to pregnant women (e.g., who should be directed to consult with a social work or counselor, who should be encouraged to attend educational classes that improve maternal-fetal health). For example, our research suggests that women who scored high on openness to experience are less likely to quit alcohol consumption in pregnancy, which may be due to these women doubting their doctor’s recommendations or evidence about the harmful effects of drinking alcohol during pregnancy. Better understanding of personality traits associated with health behaviors in pregnancy may allow for more targeted and effective health-promotion communication that improve women' abilities to exercise appropriate control over their health. Pregnant women are usually highly motivated to improve their health and a targeted health promotion communication may be more effective in helping women to understand that quitting maladaptive habits reduces health risks to their children as well as themselves [76].

## References

[1] Gupta PC, Subramoney S. Smokeless tobacco use, birth weight, and gestational age: population based, prospective cohort study of 1217 women in Mumbai, India. BMJ. 2004 6 26;328(7455):1538 10.1136/bmj.38113.687882.EB 15198947 PMC437147

[2] Pollock HA. Sudden infant death syndrome, maternal smoking during pregnancy, and the cost effectiveness of smoking cessation intervention. Am J Public Health. 2001 3;91(3):432–6. 10.2105/ajph.91.3.432 11236409 PMC1446585

[3] Tong VT, Dietz PM, Farr SL, D'Angelo DV, England LJ. Estimates of smoking before and during pregnancy, and smoking cessation during pregnancy: comparing two population-based data sources. Public Health Rep. 2013 May-Jun;128(3):179–88. 10.1177/003335491312800308 23633733 PMC3610070

[4] Wisborg K, Kesmodel U, Henriksen TB, Olsen SF, Secher NJ. A prospective study of smoking during pregnancy and SIDS. Arch Dis Child. 2000 9;83(3):203–6. 10.1136/adc.83.3.203 10952633 PMC1718455

[5] World Health Organization. WHO Recommendations for the Prevention and Management of Tobacco use and Second-Hand Smoke Exposure in Pregnancy. Geneva: WHO; 2013. 24649520

[6] Ernst M, Moolchan ET, Robinson ML. Behavioral and neural consequences of prenatal exposure to nicotine. J Am Acad Child Adolesc Psychiatry. 2001 6;40(6):630–641. 10.1097/00004583-200106000-00007 11392340

[7] Wickström R. Effects of nicotine during pregnancy: human and experimental evidence. Curr Neuropharmacol. 2007 9;5(3):213–22. 10.2174/157015907781695955 19305804 PMC2656811

[8] Henderson J, Gray R, Brocklehurst P. Systematic review of effects of low moderate prenatal alcohol exposure on pregnancy outcome. BJOG. 2007 3;114(3):243–52. 10.1111/j.1471-0528.2006.01163.x 17233797

[9] Kesmodel U, Wisborg K, Olsen SF, Henriksen TB, Secher NJ. Moderate alcohol intake during pregnancy and the risk of stillbirth and death in the first year of life. Am J Epidemiol. 2002 2 15;155(4):305–12. 10.1093/aje/155.4.305 11836194

[10] Tan CH, Denny CH, Cheal NE, Sniezek JE, Kanny D. Alcohol Use and Binge Drinking Among Women of Childbearing Age—United States, 2011–2013. MMWR Report, 2015 9; 64(37):1042–1046. 10.15585/mmwr.mm6437a3 26401713

[11] Jacobson JL, Jacobson SW. Effects of prenatal alcohol exposure on child development. Alcohol Res Health. 2002;26(4):282–6. 12875038 PMC6676687

[12] Sayal K, Heron J, Golding J, Emond A. Prenatal alcohol exposure and gender differences in childhood mental health problems: a longitudinal population-based study. Pediatrics. 2007;119:e426–434. 10.1542/peds.2006-1840 17272604

[13] Centers for Disease Control and Prevention (US) An alcohol-free pregnancy is the best choice for your baby [cited 2019 December 05] Available from: http://www.cdc.gov/ncbddd/fasd/documents/fasdbrochure_final.pdf.

[14] Sokol RJ, Delaney-Black V, Nordstrom B. Fetal alcohol spectrum disorder. JAMA. 2003 12;290(22):2996–9. 10.1001/jama.290.22.2996 14665662

[15] Curtin SC, Matthews TJ. Smoking prevalence and cessation before and during pregnancy: data from the birth certificate, 2014. Natl Vital Stat Rep. 2016;65(1):1–14. 26905977

[16] Ebrahim SH, Luman ET, Floyd RL, Murphy CC, Bennett EM, Boyle CA. Alcohol consumption by pregnant women in the United States during 1988–1995. Obstet Gynecol. 1998;92:187–92. 10.1016/s0029-7844(98)00205-1 9699749

[17] Chamberlain C, O'Mara-Eves A, Oliver S, Caird JR, Perlen SM, Eades SJ, et al Psychosocial interventions for supporting women to stop smoking in pregnancy. Cochrane Database Syst Rev. 2013 10 23;10(10):CD001055. 10.1002/14651858.CD001055.pub4 PMC4022453 24154953

[18] Schneider S, Huy C, Schütz J, Diehl K. Smoking cessation during pregnancy: a systematic literature review. Drug Alcohol Rev. 2010 1;29(1):81–90. 10.1111/j.1465-3362.2009.00098.x 20078687

[19] Andres RL, Day MC. Perinatal complications associated with maternal tobacco use. Seminars in Neonatology: SN. 2000 8;5(3):231–241. 10.1053/siny.2000.0025 10956448

[20] Terracciano A, Costa PT Jr. Smoking and the Five-Factor Model of personality. Addiction. 2004 4;99(4):472–81. 10.1111/j.1360-0443.2004.00687.x 15049747 PMC2376761

[21] Turiano NA, Hill PL, Roberts BW, Spiro A 3rd, Mroczek DK. Smoking mediates the effect of conscientiousness on mortality: The Veterans Affairs Normative Aging Study. J Res Pers. 2012 12;46(6):719–724. 10.1016/j.jrp.2012.08.009 23504043 PMC3595549

[22] Kazdin AE., editor. Encyclopedia of Psychology. Vol. 5 New York: Oxford University Press; 2000.

[23] Costa PT, McCrae RR. Revised NEO Personality Inventory (NEO-PI-R) and NEO Five-Factor Inventory (NEO-FFI). Professional Manual. Odessa, FL: Psychological Assessment Resources; 1992.

[24] Lerman C, Caporaso NE, Audrain J, Main D, Boyd NR, Shields PG. Interacting effects of the serotonin transporter gene and neuroticism in smoking practices and nicotine dependence. Mol Psychiatry. 2000 3;5(2):189–92. 10.1038/sj.mp.4000672 10822347

[25] Mroczek DK, Spiro A, Turiano N. Do Health Behaviors Explain the Effect of Neuroticism on Mortality? Longitudinal Findings from the VA Normative Aging Study. J Res Pers. 2009 8 1;43(4):653–659. 10.1016/j.jrp.2009.03.016 20161240 PMC2705907

[26] Turiano NA, Chapman BP, Gruenewald TL, Mroczek DK. Personality and the leading behavioral contributors of mortality. Health Psychol. 2015 1;34(1):51–60. 10.1037/hea0000038 24364374 PMC4103968

[27] Tate JC, Pomerleau CS, Pomerleau OF. Pharmacological and non-pharmacological smoking motives: a replication and extension. Addiction. 1994 3;89(3):321–30. 10.1111/j.1360-0443.1994.tb00899.x 8173502

[28] Almada SJ, Zonderman AB, Shekelle RB, Dyer AR, Daviglus ML, Costa PT Jr, et al Neuroticism and cynicism and risk of death in middle-aged men: the Western Electric Study. Psychosom Med. 1991 Mar-Apr;53(2):165–75. 10.1097/00006842-199103000-00006 2031070

[29] Munafo MR, Zetteler JI, Clark TG. Personality and smoking status: A meta analysis. Nicotine Tob Res. 2007 3;9(3):405–13. 10.1080/14622200701188851 17365772

[30] Rausch JL, Nichinson B, Lamke C, Matloff J. Influences of negative affect on smoking cessation treatment outcome: A pilot study. British Journal of Addiction. 1990;85:929–933. 10.1111/j.1360-0443.1990.tb03723.x 2397320

[31] Grekin ER, Sher KJ, Wood PK. Personality and substance dependence symptoms: Modeling substance-specific traits. Psychol Addict Behav. 2006 12;20(4):415–24. 10.1037/0893-164X.20.4.415 17176176

[32] Larkins JM, Sher K J. Family history of alcoholism and the stability of personality in young adulthood. Psychol Addict Behav. 2006 12;20(4):471–7. 10.1037/0893-164X.20.4.471 17176182

[33] Malouff JM, Thorsteinsson EB, Rooke SE, Schutte NS. Alcohol involvement and the Five-Factor model of personality: a meta-analysis. J Drug Educ. 2007;37(3):277–94. 10.2190/DE.37.3.d 18047183

[34] Read JP, O’Connor RM. High- and low-dose expectancies as mediators of personality dimensions and alcohol involvement. J Stud Alcohol. 2006 3;67(2):204–14. 10.15288/jsa.2006.67.204 16562402

[35] Asendorpf JB, Wilpers S. Personality effects on social relationships. J. Pers. Soc. Psychol. 1998;74:1531–44.

[36] Russell DW, Booth B, Reed D, Laughlin PR. Personality, social networks, and perceived social support among alcoholics: A structural equation analysis. J Pers. 1997 9;65(3):649–92. 10.1111/j.1467-6494.1997.tb00330.x 9327590

[37] Arai Y, Hosokawa T, Fukao A, Izumi Y, Hisamichi S. Smoking behaviour and personality: A population-based study in Japan. Addiction. 1997 8;92(8):1023–33. 9376772

[38] Kassel JD, Stroud LR, Paronis CA. Smoking, stress, and negative affect: Correlation, causation, and context across stages of smoking. Psychol Bull. 2003 3;129(2):270–304. 10.1037/0033-2909.129.2.270 12696841

[39] Wilkinson D, Abraham C. Constructing and integrated model of the antecedents of adolescent smoking. Br J Health Psychol. 2004 9;9(Pt 3):315–33. Available from: http://www.ncbi.nlm.nih.gov/books/NBK190304/pdf/TOC.pdf. 10.1348/1359107041557075 15296680

[40] Raynor D, Levine H. Associations between the five-factor model of personality and health behaviors among college students. J Am Coll Health. 2009 Jul-Aug;58(1):73–81. 10.3200/JACH.58.1.73-82 19592356

[41] Chapman S, Freeman B. Markers of the denormalisation of smoking and the tobacco industry. Tob Control. 2008 2;17(1):25–31. 10.1136/tc.2007.021386 18218803

[42] Chassin L, Presson C, Morgan-Lopez A, Sherman SJ. Deviance proneness and adolescent smoking 1980 vs. 2001: has there been a hardening of adolescent smoking? J Appl Dev Psychol. 2007;28:264–76.

[43] Stuber J, Galea S, Link BG. Smoking and the emergence of a stigmatized social status. Soc Sci Med. 2008 8;67(3):420–30. 10.1016/j.socscimed.2008.03.010 18486291 PMC4006698

[44] Gilbert DG, McClernon FJ, Gilbert BO. The psychology of the smoker. In: Bollinger CT, Fagerström KO, editors. The tobacco epidemic. Prog Respir Res [Basel] 1997;28:132–150.

[45] Hill SY, Shen S, Lowers L, Locke J. Factors predicting the onset of adolescent drinking in families at high risk for developing alcoholism. Biological Psychiatry. 2000;48(4):265–275. 10.1016/s0006-3223(00)00841-6 10960157

[46] Hill SY, Yuan H. Familial density of alcoholism and onset of adolescent drinking. J Stud Alcohol 1999;60: 7–17. 10.15288/jsa.1999.60.7 10096304

[47] Cook M, Young A, Taylor D, Bedford AP. Personality correlates of alcohol consumption. Pers Individ Dif. 1998;24:641–7.

[48] Fairbairn CE, Sayette MA, Wright AG, Levine JM, Cohn JF, Creswell KG. Extraversion and the Rewarding Effects of Alcohol in a Social Context. J Abnorm Psychol. 2015 8;124(3):660–73. 10.1037/abn0000024 25844684 PMC4595151

[49] Caligiuri PM. The Big Five personality characteristics as predictors of expatriate’s desire to terminate the assignment and supervisor-rated performance. Pers. Psychol. 2000;53, 67–88.

[50] Bogg T, Roberts BW. Conscientiousness and health behaviors: A metaanalysis of the leading behavioral contributors to mortality. Psychol Bull. 2004 11;130(6):887–919. 10.1037/0033-2909.130.6.887 15535742

[51] Kubicka L, Matejcek Z, Dytrych Z, Roth Z. IQ and personality traits assessed in childhood as predictors of drinking and smoking behaviour in middle-aged adults: A 24 year follow-up study. Addiction. 2001 11;96(11):1615–28. 10.1080/09652140120080741 11784458

[52] Malouff JM, Thorsteinsson EB, Schutte NS. The five-factor model of personality and smoking: A meta-analysis. J Drug Educ. 2006;36:47–58. 10.2190/9EP8-17P8-EKG7-66AD 16981639

[53] Vollrath M, Knoch D, Cassano L. Personality, risky health behaviour, and perceived susceptibility to health risks. Eur. J. Pers. 1999 2;13:39–50.

[54] Hakulinen C, Hintsanen M, Munafò MR, Virtanen M, Kivimäki M, Batty GD, et al Personality and smoking: individual-participant meta-analysis of nine cohort studies. Addiction. 2015 11;110(11):1844–52. 10.1111/add.13079 Epub 2015 Aug 22. ; PMCID: PMC4609271. 26227786 PMC4609271

[55] Hakulinen C, Jokela M. Alcohol use and personality trait change: pooled analysis of six cohort studies. Psychol Med. 2019 1;49(2):224–231. 10.1017/S0033291718000636 29540247

[56] Zvolensky MJ, Taha F, Bono A, Goodwin RD. Big five personality factors and cigarette smoking: a 10-year study among US adults. J Psychiatr Res. 2015 4;63:91–6. 10.1016/j.jpsychires.2015.02.008 25799395 PMC4422054

[57] Martin JL, Groth G, Longo L, Rocha TL, Martens MP. Disordered eating and alcohol use among college women: associations with race and big five traits. Eat Behav. 2015;17:149–52. 10.1016/j.eatbeh.2015.02.002 25734858

[58] Scaife JC, Duka T. Behavioural measures of frontal lobe function in a population of young social drinkers with binge drinking pattern. Pharmacol Biochem Behav. 2009; 93:354–62. 10.1016/j.pbb.2009.05.015 19497334

[59] Rush CC, Becker SJ, Curry JF. Personality factors and styles among college students who binge eat and drink. Psychol Addict Behav. 2009 23:140–5. 10.1037/a0013167 19290698

[60] Hakulinen C, Elovainio M, Batty GD, Virtanen M, Kivimäki M, Jokela M. Personality and alcohol consumption: Pooled analysis of 72,949 adults from eight cohort studies. Drug Alcohol Depend. 2015;151:110–114. 10.1016/j.drugalcdep.2015.03.008 25823906 PMC4447572

[61] Leung DY, Au DW, Lam T, Chan SS. Predictors of long-term abstinence among Chinese smokers following treatment: the role of personality traits. Asian Pac J Cancer Prev. 2013;14:5351–5354. 10.7314/apjcp.2013.14.9.5351 24175824

[62] Roberts BW, Wood D, Smith JL. Evaluating five factor theory and social investment perspectives on personality trait development. J. Res. Pers. 2005;39(1):166–84.

[63] Sampson RJ, Laub JH. Crime and deviance over the life course: The salience of adult social bonds. Am. Sociol. Rev. 1990 10;55:609–27.

[64] Beijers C, Burger H, Verbeek T, Bockting CL, Ormel J. Continued smoking and continued alcohol consumption during early pregnancy distinctively associated with personality. Addict Behav. 2014 5;39(5):980–6. 10.1016/j.addbeh.2014.01.022 24556156

[65] Maxson PJ, Edwards SE, Ingram A, Miranda ML. Psychosocial differences between smokers and non-smokers during pregnancy. Addict. Behav. 2012 2;37(2):153–159. 10.1016/j.addbeh.2011.08.011 22000409

[66] Massey SH, Lieberman DZ, Reiss D, Leve LD, Shaw DS, Neiderhiser JM. Association of clinical characteristics and cessation of tobacco, alcohol, and illicit drug use during pregnancy. Am J Addict. 2011 Mar-Apr;20(2):143–50. 10.1111/j.1521-0391.2010.00110.x 21314757 PMC3052631

[67] Yali AM, Lobel M. Coping and distress in pregnancy: an investigation of medically high risk women. J Psychosomatic Obstetr Gynecol. 1999;20(1):39–52. 10.3109/01674829909075575 10212886

[68] Dunkel Schetter C, Glynn L. Stress in pregnancy: empirical evidence and theoretical issues to guide interdisciplinary researchers. In: Contrada R, Baum A, editors. Handbook of stress science: biology, psychology, and health. Springer Publishing Company; New York, NY: 2011 pp. 321–343.

[69] Pickett KE, Wood C, Adamson J, D'Souza L, Wakschlag LS. Meaningful differences in maternal smoking behaviour during pregnancy: implications for infant behavioural vulnerability. J Epidemiol Community Health. 2008 4;62(4):318–24. 10.1136/jech.2006.058768 18339824 PMC10087306

[70] Miller GE, Culhane J, Grobman W, et al Mothers' childhood hardship forecasts adverse pregnancy outcomes: Role of inflammatory, lifestyle, and psychosocial pathways. Brain Behav Immun. 2017;65:11–19. 10.1016/j.bbi.2017.04.018 28450221 PMC5537016

[71] Miller GE, Borders AE, Crockett AH, et al Maternal socioeconomic disadvantage is associated with transcriptional indications of greater immune activation and slower tissue maturation in placental biopsies and newborn cord blood. Brain Behav Immun. 2017;64:276–284. 10.1016/j.bbi.2017.04.014 28434870 PMC5493326

[72] McCrae RR, Terracciano A, Members of the Personality Profiles of Cultures Project. Universal Features of Personality Traits From the Observer's Perspective: Data From 50 Cultures. J Pers Soc Psychol. 2005 3;88(3):547–61. 10.1037/0022-3514.88.3.547 15740445

[73] Cohen J, Statistical Power Analysis for the Behavioral Sciences. New York: NY: Routledge Academic; 1988.

[74] IBM, SPSS

[75] Cerdá M, Johnson-Lawrence VD, Galea S. Lifetime income patterns and alcohol consumption: investigating the association between long- and short-term income trajectories and drinking. Soc Sci Med. 2011 10;73(8):1178–85. 10.1016/j.socscimed.2011.07.025 Epub 2011 Aug 26. ; PMCID: PMC3185179. 21890256 PMC3185179

[76] Huckle T, You RQ, Casswell S. Socio-economic status predicts drinking patterns but not alcohol-related consequences independently. Addiction. 2010;105:1192–1202 10.1111/j.1360-0443.2010.02931.x 20456295

[77] Casetta B, Videla AJ, Bardach A, Morello P, Soto N, Lee K, et al (2017) Association between cigarette smoking prevalence and income level: a systematic review and meta-analysis. Nicotine Tob Res 19:1401–1407. 10.1093/ntr/ntw266 27679607

[78] Severson HH, Andrews JA, Lichtenstein E, Wall M, Akers L: Reducing maternal smoking and relapse: long-term evaluation of a pediatric intervention. Prev Med. 1997, 26 (1): 120–130. 10.1006/pmed.1996.9983 9010907

[79] Tong VT, Jones JR, Dietz PM, D’Angelo D, Bombard JM: Trends in smoking before, during, and after pregnancy—Pregnancy Risk Assessment Monitoring System (PRAMS), United States, 31 sites, 2000–2005. MMWR Surveill Summ. 2009, 58: 1–29. 19478726

[80] Smedberg J, Lupattelli A, Mårdby AC, Nordeng H. Characteristics of women who continue smoking during pregnancy: a cross-sectional study of pregnant women and new mothers in 15 European countries. BMC Pregnancy Childbirth. 2014 6 25;14:213 10.1186/1471-2393-14-213 ; PMCID: PMC4080751. 24964728 PMC4080751

[81] American College of Obstetricians and Gynecologists. Smoking cessation during pregnancy. Committee Opinion No. 471. Obstet Gynecol. 2010 11;116(5):1241–4. 10.1097/AOG.0b013e3182004fcd 20966731

[82] Orleans CT, Johnson RW, Barker DC, Kaufman NJ, Marx JF. Helping pregnant smokers quit: meeting the challenge in the next decade. West J Med. 2001 4;174(4):276–81. 10.1136/ewjm.174.4.276 11290688 PMC1071357

[83] Todd D. Little, Terrence D. Jorgensen, Kyle M. Lang, E. Whitney G. Moore, On the Joys of Missing Data, Journal of Pediatric Psychology, Volume 39, Issue 2, March 2014, Pages 151–162). 10.1093/jpepsy/jst048 23836191

[84] Wong M, Koren G. Bias in maternal reports of smoking during pregnancy associated with fetal distress. Can J Public Health. 2001 Mar-Apr;92(2):109–12. 10.1007/BF03404942 11338147 PMC6979636

[85] DiClemente CC, Dolan-Mullen P, Windsor RA. The process of pregnancy smoking cessation: implications for interventions. Tob Control. 2000;9 Suppl 3(Suppl 3):III16–21. 10982900 10.1136/tc.9.suppl_3.iii16 PMC1766302

[86] Hannöver W, Thyrian JR, Röske K, Grempler J, Rumpf HJ, John U, et al Smoking cessation and relapse prevention for postpartum women: results from a randomized controlled trial at 6, 12, 18 and 24 months. Addict Behav. 2009 1;34(1):1–8 10.1016/j.addbeh.2008.07.021 18804331

[87] Curry SJ, McBride C, Grothus L, Lando H, Pirie P. Motivation for smoking cessation among pregnant women. Psychol. Addict. Behav. 2001;15(2):126–32. 10.1037//0893-164x.15.2.126 11419228

[88] Magnusson A, Göransson M, Heilig M. Hazardous alcohol users during pregnancy: psychiatric health and personality traits. Drug Alcohol Depend. 2007 7 10;89(2–3):27–81. 10.1016/j.drugalcdep.2007.01.015 17363194

[89] Skagerstróm J, Chang G, Nilsen P. Predictors of drinking during pregnancy: a systematic review. J Womens Health. 2011 6;20(6):901–13. 10.1089/jwh.2010.2216 PMC3159119 21671775

